# Conductive Cotton Fabrics for Motion Sensing and Heating Applications

**DOI:** 10.3390/polym10060568

**Published:** 2018-05-23

**Authors:** Mengyun Yang, Junjie Pan, Anchang Xu, Lei Luo, Deshan Cheng, Guangming Cai, Jinfeng Wang, Bin Tang, Xungai Wang

**Affiliations:** 1 Ministry of Education, Key Laboratory of Textile Fiber & Product, Wuhan Textile University, Wuhan 430073, China; mengyun_yang@163.com (M.Y.); 13071278863@163.com (J.P.); acxu@wtu.edu.cn (A.X.); luolei891123@126.com (L.L.); chengcds@163.com (D.C.); jinfeng.wang@deakin.edu.au (J.W.); xungai.wang@deakin.edu.au (X.W.); 2 Institute for Frontier Materials, Deakin University, Geelong, Victoria 3216, Australia

**Keywords:** conductive fabric, electromechanical performance, strain sensing, electric heating fabric

## Abstract

Conductive cotton fabric was prepared by coating single-wall carbon nanotubes (CNTs) on a knitted cotton fabric surface through a “dip-and-dry” method. The combination of CNTs and cotton fabric was analyzed using scanning electron microscopy (SEM) and Raman scattering spectroscopy. The CNTs coating improved the mechanical properties of the fabric and imparted conductivity to the fabric. The electromechanical performance of the CNT-cotton fabric (CCF) was evaluated. Strain sensors made from the CCF exhibited a large workable strain range (0~100%), fast response and great stability. Furthermore, CCF-based strain sensors was used to monitor the real-time human motions, such as standing, walking, running, squatting and bending of finger and elbow. The CCF also exhibited strong electric heating effect. The flexible strain sensors and electric heaters made from CCF have potential applications in wearable electronic devices and cold weather conditions.

## 1. Introduction

Flexible conductive materials have attracted considerable attention recently due to their potential applications in wearable displays, electronic sensors for human motion and electrically driven heaters [1,2,3,4,5,6,7]. The most common flexible sensors are generally fabricated using electrically conductive metal nanoparticles or nanowires, metal thin films, carbon nanotubes and graphene [8,9,10,11,12,13,14,15]. Although these sensors are electrically conductive and have high sensitivity, they have a small range of workable strain, which limits their practical applications. Some key factors need to be considered in designing strain sensors, such as a large strain range to monitor the human motion, rapid recoverable deformation, high sensitivity (high gauge factor (GF)) and fast response [16]. It is still a challenge to prepare strain sensors with a large workable strain range and high sensitivity.

Conductive textile materials (CTMs) have been widely used as flexible wearable devices because of their light weight, good flexibility, high stretchability and recoverable deformation [17,18,19,20,21,22]. Moreover, the CTMs possess high strength, good tear resistance, and excellent flexibility and comfort attributes. The response mechanism of CTMs-based strain sensors is that the resistance changes with stretching, which can be explained by changes in the fabric’s geometric structure and associated contact points between fibers and yarns within the structure. A number of strategies have been developed to prepare CTMs with sensing performance [21,23,24,25,26,27,28,29,30,31,32]. For example, reduced graphene oxide was combined with cotton to fabricate flexible sensing fabrics [17,20]. Nevertheless, these sensors from ordinary cotton fabrics had a relatively small strain range. Flexible strain sensors were also prepared by combining elastic polymers with carbonized cotton or silk fabrics [33,34,35]. The obtained sensors exhibited excellent sensitivity and a large strain sensing range. However, the carbonization process destroyed the structure of fabrics, which led to the loss of their original mechanical properties. Novel approaches are desirable to realize the preparation of flexible sensors with a wide strain range and intrinsic mechanical properties of fabrics. Carbon nanotubes (CNTs) have been widely used as electrical conductive materials owing to their high surface area, low electrical resistance, low mass density and high stability. Modification of textiles with CNTs can give conductivity to the substrate materials [4,36,37,38,39,40,41]. Some research works have reported on the CNT-coated cotton yarn and fabrics [42,43,44,45]. Although these CNT-coated cotton yarns and fabrics had good electrical conductivity, their elasticity and deformability were limited due to the weaving structure and material type, which limited the applications of the conductive fabrics in wearable items. Herein, we used cotton elastic fabrics to fabricate wearable fabrics that have a large strain range and good recoverable deformation. CNTs have been coated on yarns of polyurethane (PU) and cotton to develop flexible wearable devices [46,47]. However, yarns are one-dimensional textile materials, which limits their application in wearable electronic devices.

In this study, we fabricated flexible fabric strain sensors by treating elastic knitted cotton fabrics in a CNT suspension using the “dip-and-dry” method. The surface morphology, chemical structures and electrical conductivity of the CNT modified fabrics were characterized. The electromechanical performance and strain sensing properties of the CNT-cotton fabric were investigated. Human motion was monitored by the obtained flexible strain sensor. In addition, the CNT-cotton fabric displayed excellent electric heating effect.

## 2. Experimental

### 2.1. Materials

Knitted cotton fabrics (170 g/m^−2^) consisting of 97% cotton and 3% PU were kindly provided by a spinning and weaving research group from Wuhan Textile University (Wuhan, China). The elastic knitted fabrics were made from cotton/PU core spun composite yarns with a linear density of 14 tex. The fabric wale density and course density were 125/5 and 85/5 cm, respectively. High-purity, single-wall CNTs were purchased from Nanjing XFNANO Materials Tech Co., Ltd., Nanjing, China.

### 2.2. Preparation of CNT-Cotton Fabric

**Figure 1:**
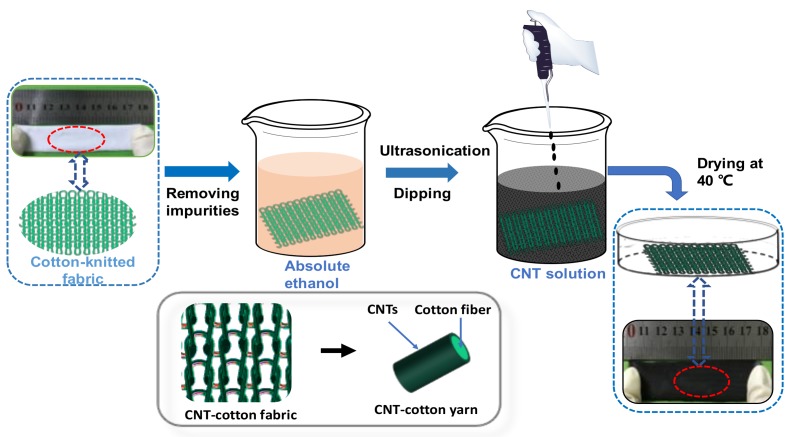
Preparation process of CNT-cotton fabrics.

The CNT-cotton fabric (CCF) was prepared by a “dip-and-dry” method, which is illustrated in Figure 1. Neat elastic knitted cotton fabrics were soaked in ethanol for 30 min and then washed thoroughly with deionized water. A stable CNT suspension was obtained through dispersing the CNTs in water followed by 15 min sonication at room temperature. The cotton fabric (4 cm × 4 cm) was immersed in CNT suspension with different concentrations (0.025, 0.050, 0.075, 0.10 wt %) and kept in solution for 20 min at room temperature under sonication. Subsequently, the fabric was dried in an oven at 40 °C. The color of the fabric changed from white to black after the adsorption of CNTs. Different cycles of “dip-and-dry” were performed to obtain the CCF.

### 2.3. Instruments

Scanning electron microscopy (SEM) was carried out by a TESCAN MIRA3 field emission SEM. A Raman microscope system (Renishaw plc, Wotton-under-Edge, UK) was used to record the Raman scattering spectra of the fabric samples. A 50/N.A. 0.75 objective and a 785-nm near-IR diode laser excitation source (500 mW, 10%) were used in all measurements. Raman signals were collected using a mounted CCD camera with integration time of 10 s by single scan. An Instron Model 5566 Materials Testing System (Norwood, MA, USA) was used for testing of the mechanical properties. The fabric samples with a width of 20 mm were prepared and their wale direction strength was tested at a gauge length of 100 mm. The changes in electric resistance of fabric samples at different strain levels were recorded using a self-built fabric dynamic resistance tester. The electric heating features of fabrics were measured by a thermocouple and an infrared thermal camera (FLIR ONE Pro, Wilsonville, OR, USA).

### 2.4. Durability Test to Washing

Durability to water washing of the obtained CCF was tested in accordance with the AATCC Test Method 61-2006. The washing procedure was performed using a standard washing machine (Model SW-12AII, Wenzhou Darong Textile Instrument Co., Ltd., Wenzhou, China). The CCF with a size of 5 cm × 10 cm was washed in a rotating closed canister containing 200 mL of detergent aqueous solution (0.37 wt %) and 10 stainless steel balls. The electrical conductivity of CCF was assessed after the water washing cycles.

## 3. Results and Discussion

### 3.1. Fabrication and Characterization of CNT-Cotton Fabric

**Figure 2:**
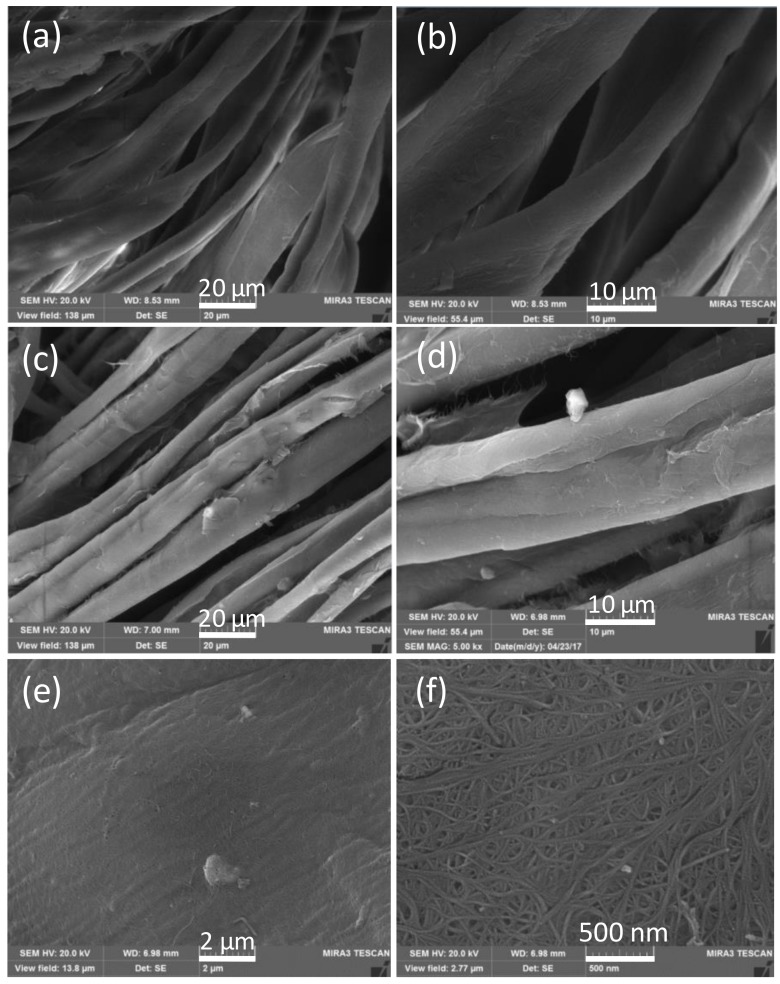
SEM images of (**a**,**b**) pristine cotton fabric and (**c**–**f**) CNT-cotton fabrics at different magnifications.

Figure 2 displays the SEM images of different fabric samples. The surface of the pristine cotton fabric was smooth without visible impurities (Figure 2a,b). After treatment with CNTs, laminar layers were clearly observed affixing to the surface of cotton fibers (Figure 2c,d), which indicates that CNTs were successfully adsorbed onto the fabrics. The magnified SEM images (Figure 2e,f) show that the overlapped nanotubes formed continuous layers on the fiber surface. The results suggest that the CNTs combined effectively with cotton fibers. The energy dispersive spectroscopy (EDS) patterns were recorded to analyze the main elements content of the cotton fabric and CCF (Figure S1 and Table S1 in supplementary material). The contents of C and O elements in the pristine cotton fabric were calculated to be 48.67 and 51.33 wt %, respectively. Compared with pristine cotton fabric, a higher C element content was found for CCF, which increased to 59.04 wt %, while a lower O element content of 40.96 wt % was observed for CCF. The obvious increase of C element in CCF further proved that CNTs were adhered to the surface of the elastic fabric.

**Figure 3:**
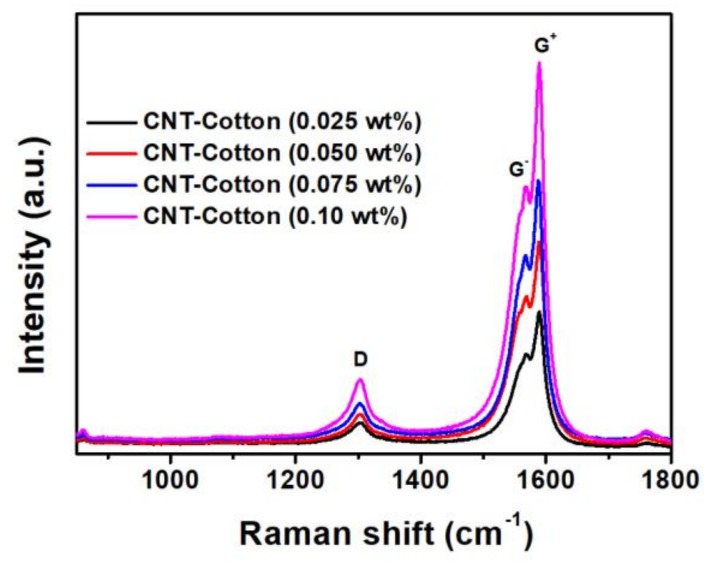
Raman scattering spectra of the elastic cotton fabrics treated with different concentrations of CNTs.

Raman scattering spectroscopy was also employed to evaluate the CNTs coating on fabrics. Three Raman bands at 1303, 1568 and 1590 cm^−1^ appear in the Raman scattering spectra of the CNTs treated elastic cotton fabrics (Figure 3) [48,49,50,51]. These three Raman bands are attributed to the characteristic Raman spectral features of single-wall CNTs, including D band (1303 cm^−1^), G^−^ band (1568 cm^−1^) and G^+^ band (1590 cm^−1^). The D band is related to the disorder-induced feature of the nanotubes. The G band of single-wall CNTs splits into two features (G^−^ band and G^+^ band), which are both first-order Raman modes. Generally, the G^−^ band, as a lower-frequency radial breathing mode, is direct Raman spectral evidence for the presence of single-wall CNTs. The Raman spectroscopic analysis further proves that the CNTs successfully combined with cotton fabrics. Moreover, it was found that the intensity of Raman bands of the treated fabrics increased as the concentration of CNTs increased, which implies that a high concentration of CNT can improve its loading amount on elastic fabrics.

### 3.2. Electrical Conductivity of CNT-Cotton Fabric

**Figure 4:**
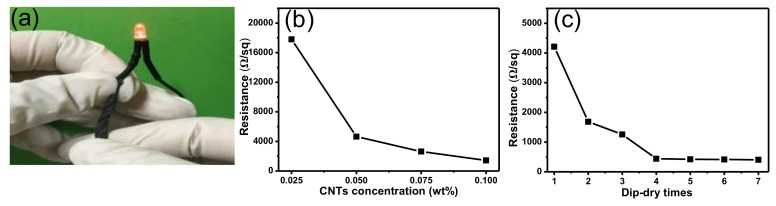
(**a**) A twisted CCF was used as a conductive wire for a light-emitting diode; (**b**) electrical resistivity of CNT-cotton fabrics corresponding to different CNT concentrations (one dip-dry cycle); and (**c**) different numbers of dip-dry cycles (0.05 wt % of CNT concentration).

A light-emitting diode (LED) was lit up by wiring a twisted CCF with a power supply (Figure 4a), verifying that CCF is electrically conductive. The loading amount of CNT on the cotton fabrics has a direct impact on the electrical conductivity of the obtained fabric. The effect of CNT concentration on the electrical conductivity of the obtained CCF was investigated as shown in Figure 4b. The pristine cotton fabrics have an extremely high surface electrical resistance (~10^9^ Ω/sq). It can be seen that the surface resistance decreased significantly when the CNT concentration increased from 0.025 to 0.1 wt %. The average surface electrical resistance of CCF reached 1435 Ω/sq when the CNT concentration was 0.1 wt %. In addition to CNT concentration, the number of dip-dry cycles also has a remarkable effect on the electrical resistance of CCF. The resistance of CCF was 4211 Ω/sq after the first dip-dry cycle. The resistance of CCF decreased greatly with an increasing number of dip-dry cycles and decreased to 439 Ω/sq after four dip-dry cycles (Figure 4c). On the other hand, the surface resistance of CCF tended to be stable when the number of dip-dry cycles was more than four, which may be caused by the adsorption of CNTs reaching saturation on the fabric.

### 3.3. Durability of the CNT-Cotton Fabric to Washing

**Figure 5:**
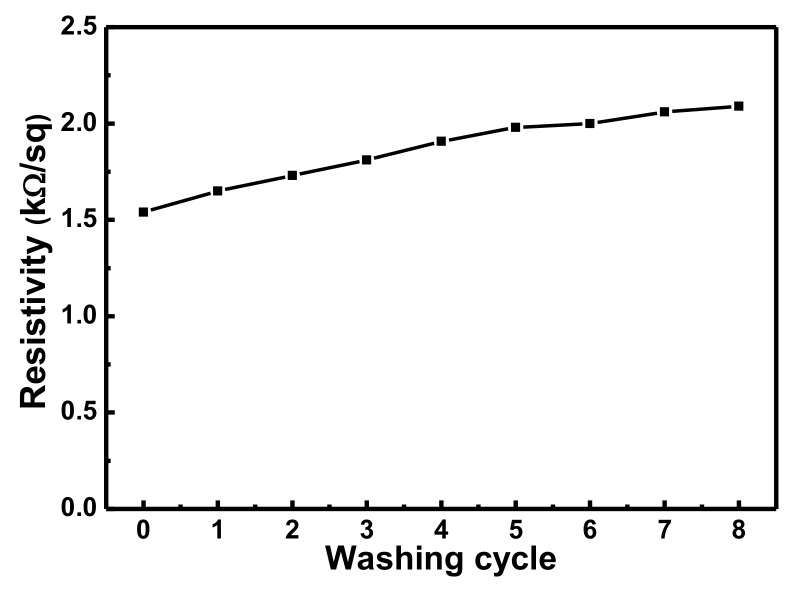
The resistivity change of CNT-cotton after different numbers of washing cycles.

To evaluate the washability of the as-prepared conductive fabric, the electrical resistance of CCF after the washing test was measured and the effect of the washing cycles on the electrical conductivity was analyzed. As shown in Figure 5, the electrical resistance increased slightly in the first few washing cycles, from 1.55 kΩ/sq after the first cycle to 1.81 kΩ/sq after three cycles. After eight washing cycles, the electrical resistance still remained lower than 2.1 kΩ/sq, indicating that the number of washing cycles did not significantly impact the conductivity of the CCF.

### 3.4. Mechanical Properties of CNT-Cotton Fabric

**Figure 6:**
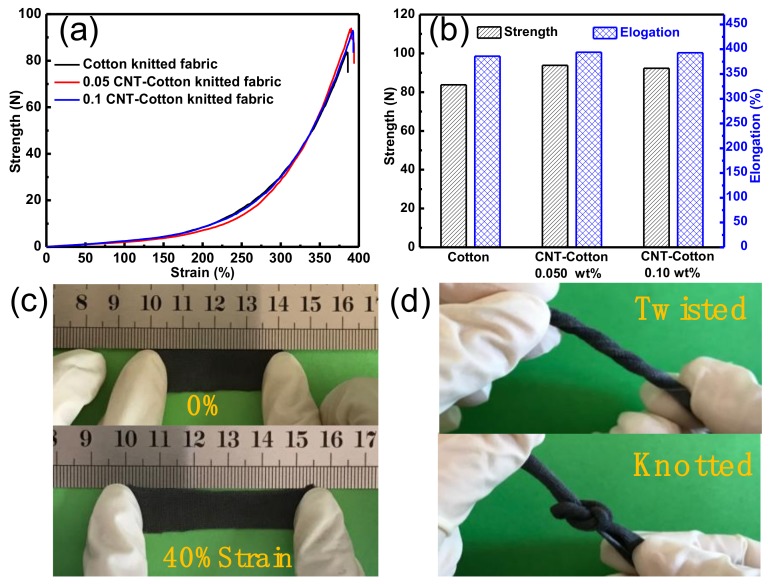
(**a**) Strength-elongation curves and (**b**) mechanical testing values for neat knitted cotton fabric and CCFs. Photographs of (**c**) CCF before and after the loading of a tensile strain of 40%, and (**d**) a twisted and knotted CCF.

In order to investigate the influence of CNT treatment on the mechanical properties of cotton fabric, tensile measurement of pristine fabric and CNT-treated cotton fabrics were performed. Figure 6a shows typical strength-elongation curves of pristine fabric and the cotton fabric treated with different concentrations of CNTs (0.05 and 0.10 wt %). The strength-elongation curves of the three samples are similar in profile, implying that the treatment with CNTs slightly improved the mechanical properties. As seen from Figure 6b, the breaking strength of the fabric slightly increased from 83.8 to 93.8 N when the fabrics were coated by CNTs (0.05 wt %). We suggest that the existence of the CNTs may increase the binding strength between fibers and yarns, leading to improved fabric strength. The breaking elongation of fabrics showed no notable changes after CNT treatment (close to 400%). The tensile test results reveal that the treatment with CNTs improved the mechanical properties of the elastic fabrics. Moreover, CCF exhibited excellent elasticity and flexibility, which enable it to be stretched, twisted and knotted (Figure 6c,d). The excellent mechanical features, including breaking strength, elongation and flexibility, pave the way for practical applications of CCFs.

### 3.5. Electromechanical Performance of the CNT-Cotton Fabric

**Figure 7:**
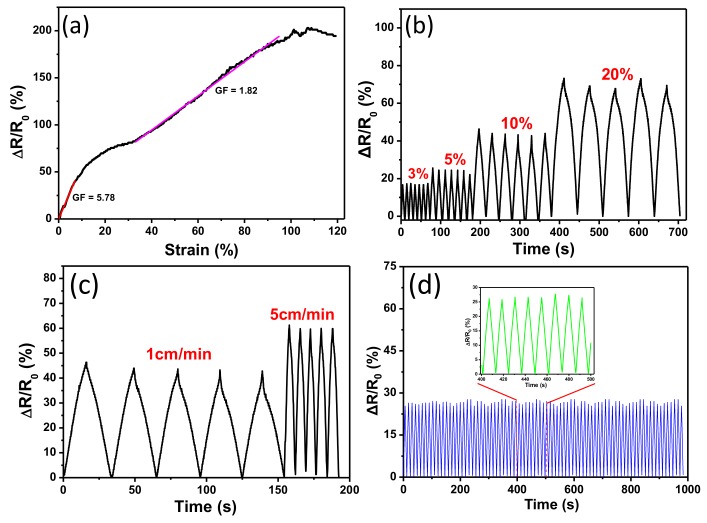
(**a**) Relative resistance change (∆*R*/*R*_0_) as a function of tensile strain of CCF; (**b**) relative resistance variation (∆*R*/*R*_0_) versus cyclic tensile strain of 3%, 5%, 10% and 20%; (**c**) resistance change under cyclic stretching-releasing with a strain of 10% at different loading speed; (**d**) the durability test of CCF under cyclic tensile.

The wale direction of knitted fabric has higher elasticity than the course direction, which contributes to higher sensitivity and resistance changes of conductive fabric under stretching, resulting from the deformation of the weaving structures of fabrics [52]. The changes in relative resistance (∆*R*/*R*_0_) of the CCF were plotted against the strain range from 0% to 120% in wale direction (Figure 7a), where *R*_0_ and ∆*R* represent the initial resistance before stretching and corresponding resistance change, respectively. It can be seen that the CCF displays a linearly monotonic increase in resistance with a tensile strain up to 100%. The results reveal that the CCF has a very wide strain sensing range. The gauge factor was calculated to be 5.78 in a strain range from 0% to 10%, suggesting that the CCF has good sensing performance in a low strain range. Although the gauge factor decreased when the strain was more than 10%, the gauge factor still reached 1.82 in the strain range of 40~100%. In our previous research, graphene-coated elastic nylon fabric was used as a sensing fabric and exhibited high sensitivity [52]. However, the strain sensing range of the graphene-coated elastic nylon fabric was only 0~33%, which limits its applications in monitoring large motion.

Figure 7b shows the relative resistance change of a CCF-based strain sensor under stretching-releasing cycles carried out with different strains but the same loading speed. The relative resistance change increased as the strain increased, which was consistent with the results shown in Figure 7a. The CCF under different strain displayed stable ∆*R*/*R*_0_ with good repeatability, revealing that the CCF could be used in practical strain sensing applications with a reliable response. The loading rate of strain (1 and 5 cm/min) also influenced the resistance change even though the stain was kept consistent (10%) (Figure 7c). The relative resistance change slightly increased as we increased the loading speed, which may be due to the limited immediate structure response of CCF at a high loading speed [52]. Furthermore, long-term stability and repeatability are of great importance for strain sensors to be used in practice. As shown in Figure 7d, the CCF strain sensor exhibited a highly stable and repeatable relative resistance change under 100 stretching-releasing cycles with a strain of 5% at a loading speed of 1 cm/min, which shows the CCF structure is very stable without obvious damage under cyclic loading of tensile strain.

### 3.6. Strain Sensing Performance of the CNT-Cotton Fabric

**Figure 8:**
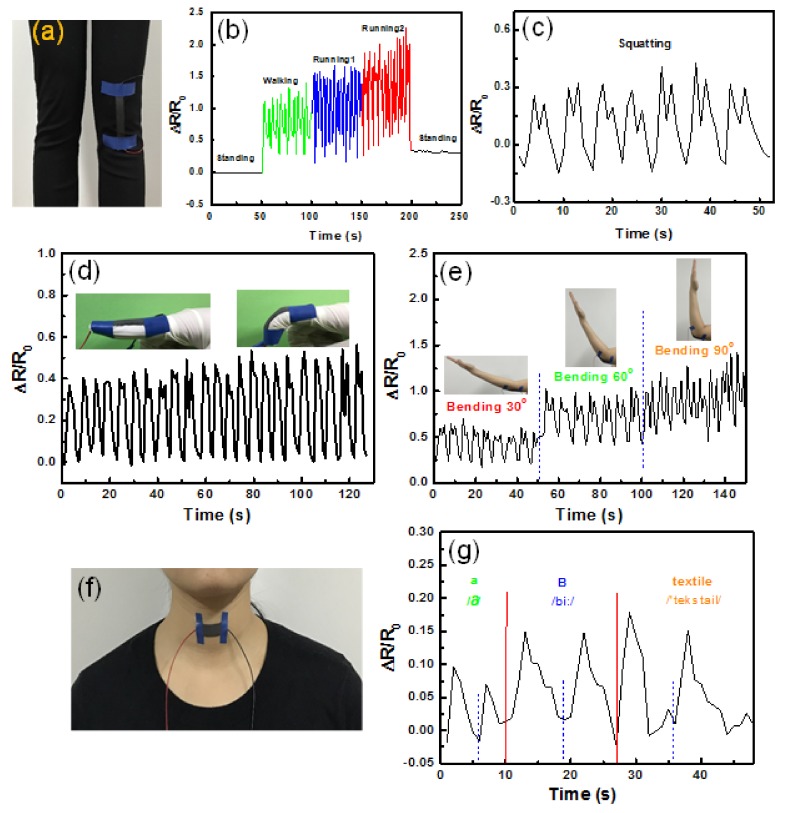
Detection of various human motions using CCF. (**a**) Photograph of CCF attached directly on a knee; (**b**,**c**) responsive curves of CCF on the knee under motions of standing, walking, running and squatting; (**d**) responsive curve of CCF on finger; (**e**) responsive curve of CCF on the elbow under cyclic bending; (**f**) photograph of CCF attached on the throat; (**g**) responsive curves of CCF when the wearer speaks “a,” “B” and “textile.”

To investigate the performance of CCF as an activity monitoring sensor, the CCF was used to monitor human motion in real time. The CCF was attached directly on a knee (Figure 8a). The resistance of CCF was collected and analyzed when the knee performed different motions, including standing, walking, running and squatting. The corresponding real-time electrical resistance change curves were shown in Figure 8b,c. It can be found that the various knee-related motions are easily discriminated by the CCF sensor. Figure 8d shows that the CCF was affixed on a finger for detecting a minor strain from bending a finger. The bending motions of the finger were precisely monitored by recording the resistance change of CCF. The changes in resistance can reflect the upward and downward movements of the finger. The cyclic bending of an elbow was also precisely tracked by CCF (Figure 8e). The intensity of curve of ∆*R*/*R*_0_ increased as the elbow bending, and then returned to the initial level after the elbow restored to its original status. Meanwhile, increasing bending angle resulted in a further increase of CCF resistance.

In addition to detecting human motion, the CCF can also promptly and accurately capture subtle physiological signals, such as phonation. CCF was fixed at a volunteer’s throat to detect the behavior of the vocal cords (Figure 8f). When “a,” “B” and “textile” were pronounced two times, a unique waveform could be easily identified, which clearly indicates that CCF possesses potential for identification of various pronunciations (Figure 8g).

### 3.7. Electric Heating Performance of the CNT-Cotton Fabric

**Figure 9:**
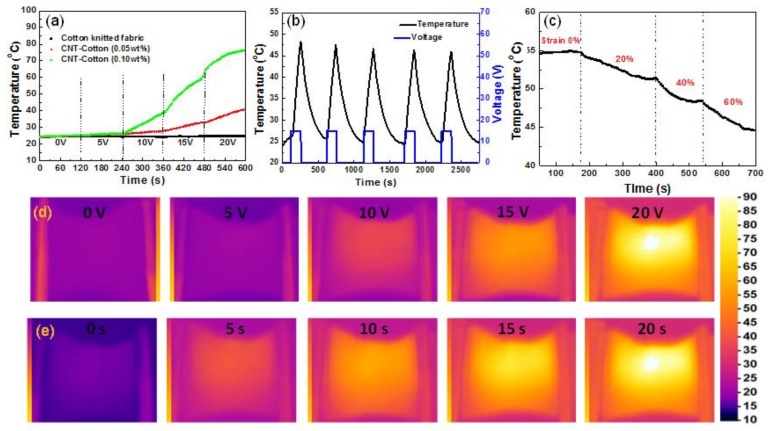
(**a**) Evolution of temperature of the CCF at different voltages; (**b**) temperature response of CCF at a cyclic voltage of 20 V; (**c**) time-dependent temperature curves of the CCF under different strains at 15 V; infrared thermal images of the CCF: (**d**) 20 s at different voltages; and (**e**) 20 V for different power-on periods.

CCF exhibited significant electric heating performance under static and dynamic states. The electric heating performance of original CCF (1 cm × 3 cm) was investigated. The time-dependent temperature curves under different voltages were recorded to characterize the electric heating behavior of CCF (Figure 9a). There was a slightly temperature change if the electric voltage was less than 5 V. The temperature of fabric surface changed dramatically when the electric voltage was higher than 5 V. The surface temperature of CCF reached 78 °C at 20 V within 2 min. The temperature of CCF increased with the increase of applied voltage, due to the Joule effect. Meanwhile, the CCF treated with a higher concentration of CNTs showed a higher surface temperature when voltage and power-on time were kept constant. It should be also noted that the pristine cotton did not exhibit any electro-heating effect even though the electricity voltage was as high as 20 V. The heating/cooling cycling tests were conducted to investigate the heating stability of the CCF. Figure 9b presents the evolution of CCF temperature at a cyclic voltage of 15 V. The maximum temperature was around 48 °C and it maintained even after five cycles, revealing the superior stability and repeatability of the CCF under working voltage. The stable heating performance of an electric heater under tensile strain is important for their application as wearable devices. The temperature of CCF under dynamic stepwise strains from 0% to 60% at a constant voltage of 15 V was recorded. As can be seen in Figure 9c, the CCF showed significant electro-heating conversion effect under large strain and the electric heating performance of the CCF deteriorated slightly corresponding to the large strain. The surface temperature of CCF still reached to 45 °C under 60% strain.

The electric heating feature of the CCF was further analyzed by applying various voltages to the samples. Figure 9d,e display the corresponding thermal images of CCF obtained using an infrared thermal camera. The electrical energy was converted into heat to gradually raise the CCF temperature: 20 V of power supply resulted in a high temperature of 90 °C on the fabric surface within 20 s. In addition, the temperature of CCF increased as we increased the power-on time when the voltage was kept at 20 V (Figure 9e). These results demonstrate that the CCF has an excellent converting effect from electricity to heating.

## 4. Conclusions

In summary, flexible wearable strain-sensing fabric was prepared using elastic knitted cotton and carbon nanotubes (CNTs) through a simple “dip-and-dry” process. Raman spectroscopy and SEM characterization confirm that the CNTs were coated onto the surface of the cotton fabric. The tensile data suggest that the combination of CNTs improved the mechanical properties of the CNT-cotton fabric (CCF). The CCF-based strain sensor exhibited a wide strain-sensing range, fast response and great stability. Furthermore, this CCF-based strain sensor enabled real-time monitoring of different human motions, including standing, walking, running, squatting, and bending of finger and elbow. Moreover, CNT coating on the surface of a fabric endowed the treated fabric with excellent electric heating features. CCF shows excellent performance as a flexible strain sensor and electric heater, which gives it great potential in wearable electronics applications.

## Supplementary Materials

The supplementary materials are available online at http://www.mdpi.com/2073-4360/10/6/568/s1.
