# Supplementary material for: Conductive Cotton Fabrics for Motion Sensing and Heating Applications
Source: Polymers (Basel). 2018 May 23;10(6):568. doi: 10.3390/polym10060568 (PMC6404225; doi:10.3390/polym10060568)
Supplement: Supplementary file 1 [file polymers-10-00568-s001.pdf]

Supporting information

Conductive Cotton Fabrics for Motion Sensing and Heating Applications

Mengyun Yang [1], Junjie Pan [1], Anchang Xu [1], Lei Luo [1], Deshan Cheng [1], Guangming Cai [1,*], Jinfeng Wang [1,2], Bin Tang [1,2,*] and Xungai Wang [1,2]

1. Ministry of Education, Key Laboratory of Textile Fiber & Product, Wuhan Textile University, Wuhan 430073, China; mengyun_yang@163.com (M.Y.); 13071278863@163.com (J.P.); acxu@wtu.edu.cn (A.X.); luolei891123@126.com (L.L.); chengcds@163.com (D.C.); jinfeng.wang@deakin.edu.au (J.W.); xungai.wang@deakin.edu.au (X.W.)
2. Institute for Frontier Materials, Deakin University, Geelong, Victoria 3216, Australia

* Correspondence: guangmingcai2006@163.com (G.C.); bin.tang@deakin.edu.au (B.T.); Tel.: +86-27-593-67572 (G.C.); +61-3-522-73374 (B.T.)

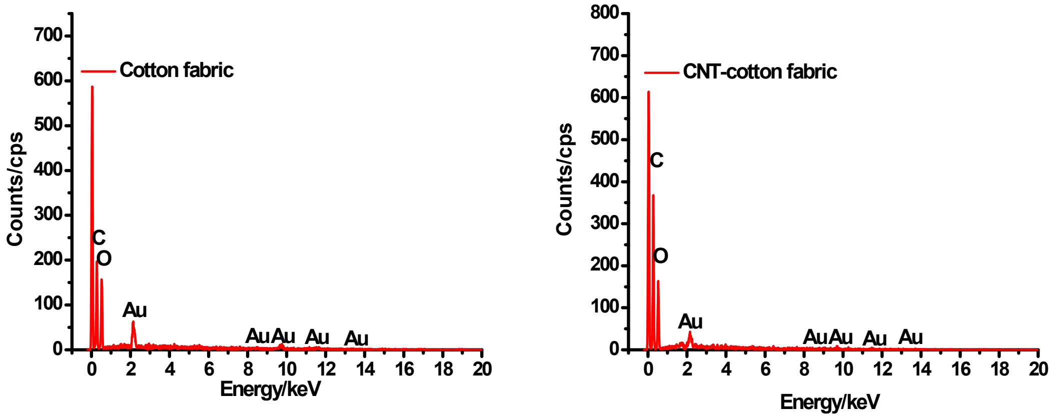

Figure S1. The EDS of CNT-cotton fabrics.

Table S1. The C and O content of cotton and CNT-cotton fabric.

Element	Cotton fabric (wt %)	CNT-cotton fabric (wt %)
C	48.67	59.04
O	51.33	40.96
